# Preferences for AI-Enabled Health Care Technologies: Systematic Review of Discrete Choice Experiments and Reporting Quality Assessment Using the DIRECT Checklist

**DOI:** 10.2196/103197

**Published:** 2026-08-10

**Authors:** Xinyue Zhang, Shimeng Liu, Yangchen Ji, Yingyao Chen

**Affiliations:** 1 School of Public Health, Fudan University, No.130 Dongan Road, Xuhui District, Shanghai, 200032, China, 86 13046006196; 2 National Health Commission Key Laboratory of Health Technology Assessment, Fudan University, Shanghai, China

**Keywords:** artificial intelligence, discrete choice experiments, health preference, DIRECT checklist, DIscrete choice experiment REporting ChecklisT, reporting quality

## Abstract

**Background:**

AI is increasingly being integrated into health care, making it important to understand stakeholder preferences for AI-enabled technologies. Although discrete choice experiments (DCEs) are widely used to elicit preferences, evidence on preference attributes, willingness to pay (WTP), and reporting quality in AI-related DCEs has not been systematically synthesized.

**Objective:**

This systematic review aims to synthesize stakeholder preferences for AI-enabled health care technologies elicited through DCEs and assess reporting quality using the DIRECT (DIscrete choice experiment REporting ChecklisT). The goal is to identify key preference attributes and methodological gaps to guide the development of AI technologies aligned with real-world needs.

**Methods:**

This systematic review was conducted in accordance with PRISMA (Preferred Reporting Items for Systematic Reviews and Meta-Analyses) guidelines. We systematically searched PubMed, Embase, Web of Science, Scopus, the Cochrane Library, and the International Health Technology Assessment (HTA) Database from database inception to March 2026. We included studies reporting original DCE data on AI-enabled health care technologies. Study characteristics, including experimental design features and econometric modeling approaches, were extracted and summarized descriptively. Attributes were systematically categorized using a structured framework informed by established HTA taxonomies. Stakeholder preference evidence was synthesized narratively. In addition, DCE design characteristics, preference outcomes, WTP estimates, and reporting quality indicators were extracted and synthesized. Reporting completeness was assessed using the DIRECT checklist.

**Results:**

Twenty-seven studies (28 DCEs) involving patients, clinicians, and the public were included, covering AI applications in diagnosis, screening, treatment, disease management, and decision support. Across the included studies, a total of 163 attributes were identified across 5 domains, and preference outcomes were synthesized at the level of 30 application contexts. Usability domain attributes were most frequently included, accounting for 36.81% (60/163) of all attributes, with presentation format being the most frequently reported usability attribute (27/60, 45.00%), but it was often rated least important (9/30, 30.00%). In contrast, performance attributes, especially effectiveness (17/30, 56.67%), were most often ranked as most important. Preference heterogeneity varied by context, with performance attributes dominating in clinical applications and usability attributes being more prominent in decision support. Conditional and mixed logit models were commonly used, while scale heterogeneity was rarely explored. Average reporting completeness was high (mean 84.47%, SD 11.49%), although design effects (10/27, 37.04%) and randomization (14/27, 51.85%) were inconsistently reported.

**Conclusions:**

DCE evidence suggests a mismatch between commonly included attributes and stakeholder priorities. Effectiveness is generally a key determinant of preferences, although preference structures are context dependent. Usability and performance attributes vary in importance across AI applications. Although overall reporting completeness was high, design effects and randomization were inconsistently reported. These findings highlight the need to align attribute selection with decision contexts and improve transparency in study design and reporting, which may enhance the interpretability of DCE evidence and support the development of AI technologies reflecting real-world health care needs.

## Introduction

AI technologies are increasingly being integrated into health care systems and are transforming the delivery of medical services [1]. Evolving from early rule-based systems to modern approaches such as machine learning, deep learning, and natural language processing, AI can analyze large data sets to identify patterns and relationships beyond human perception, with recent advances such as large language models further extending its capabilities [2]. Their applications have expanded beyond early use in diagnostic support to a wide range of areas, including disease screening, treatment decisions, chronic disease management, medication consultation, and remote health monitoring [3]. AI-enabled technologies are developing rapidly and are becoming more embedded in health care decision-making and service delivery. Although their clinical use remained limited in 2021, with only a small proportion of AI technologies implemented in practice [4], adoption has expanded rapidly in recent years. By March 2024, approximately 79% of health care organizations reported using AI technologies in the Microsoft-International Data Corporation study [5]. At the same time, growing evidence indicates that AI-based approaches can achieve performance and convenience comparable to, or even exceeding, those of traditional methods [6,7].

As AI increasingly permeates health care, a range of concerns has emerged. Stakeholders worry that AI systems cannot fully replicate clinician decision-making, as they may overlook patient cognitive status, quality of life, and individual preferences [8]. Additional concerns include the lack of transparency and explainability of algorithms, potential errors or malfunctions, overreliance on technology that could dehumanize patient care [9], and data privacy and security issues and other potential challenges [10]. These multidimensional characteristics may influence trust, acceptance, and ultimately the adoption of AI-enabled health care services. Understanding how these characteristics are perceived and which attributes are most valued by patients, professionals, and other stakeholders is essential for promoting the adoption of AI in health care and ensuring that AI-enabled technologies are better aligned with real-world needs, especially for patients and implementation contexts.

Discrete choice experiments (DCEs), grounded in random utility theory, are widely used to elicit preferences by asking individuals to choose between hypothetical alternatives that vary across multiple attributes and levels [11,12]. By requiring individuals to select between alternatives, DCEs allow researchers to quantify trade-offs between service characteristics and have been shown to approximate real-world decision-making, correctly predicting more than 93% of choices [13]. Given the multidimensional nature of AI-enabled health care technologies, this approach is particularly useful for identifying the attributes that stakeholders value most when evaluating such innovations. Beyond preference estimation, the validity and comparability of DCE evidence depend on transparent reporting of methodological procedures. Incomplete reporting of key elements, including attribute development, experimental design, randomization, and econometric modeling, may limit the credibility, reproducibility, and synthesis of findings [14,15]. This issue is especially relevant in AI-enabled health care, where the “black-box” nature of AI systems has heightened concerns regarding transparency, trustworthiness, and real-world implementation [16]. Transparent reporting is therefore essential for interpreting preference evidence and supporting evidence synthesis.

In recent years, an increasing number of studies have applied DCEs to investigate preferences for AI applications in health care across different clinical contexts. However, these studies vary considerably in their attribute development, experimental design, sample characteristics, and analytical approaches, and their methodological and reporting quality has not yet been systematically assessed. Two studies summarized stakeholder views on AI or DCEs in health care. Vo et al [17] synthesized qualitative and survey-based evidence on attitudes toward AI use in health care but did not examine attributes derived from DCEs that capture stakeholder trade-offs. Har et al [18] reviewed DCEs examining public preferences for emerging cancer screening technologies, but the review focused on screening modalities rather than AI-enabled health care technologies.

Therefore, our study aims to systematically review DCEs evaluating AI-enabled health care technologies with two objectives: (1) synthesizing evidence on stakeholder preferences by identifying attributes revealed through choice-based trade-offs, thereby informing the design and implementation of AI technologies that better align with user needs and real-world contexts, and (2) assessing reporting quality using the DIRECT (DIscrete choice experiment REporting ChecklisT) to identify methodological and reporting gaps and inform improvements in future DCE research. By providing a consolidated evidence base of user priorities and evaluating the rigor of existing literature, this review seeks to inform the design and implementation of AI health care solutions that are better aligned with stakeholder needs and real-world clinical contexts.

## Methods

The review methods followed the PRISMA (Preferred Reporting Items for Systematic Reviews and Meta-Analyses) guideline. Our study was reported per the PRISMA guidelines 2020 (Checklist 1), and the review protocol has been registered with PROSPERO (International Prospective Register of Systematic Reviews; CRD420261333555).

### Ethical Considerations

This work required no ethical approval.

### Data Sources

Studies were identified through standard literature searches of PubMed (MEDLINE), Embase, Scopus, Web of Science, the Cochrane Library, and the International Health Technology Assessment (HTA) Database from database inception to March 19, 2026.

### Search Strategy

Keywords and related terms for “discrete choice experiment” and “artificial intelligence,” including their synonyms and variations, were incorporated into the search strategy (the full search strategy is provided in Table S1 Multimedia Appendix 1). Only studies published in English were included.

### Study Selection

After removing duplicate studies using EndNote, 2 reviewers (XZ and YJ) independently screened the titles and abstracts of an initial set of 50 articles to assess eligibility. Discrepancies were discussed until consensus was reached, leading to a refinement of the inclusion and exclusion criteria. The remaining articles were then screened independently by XZ and YJ, with any disagreements resolved by a third reviewer (SL). Studies were included if they (1) used a DCE methodology; (2) evaluated AI-enabled technologies; in this study, AI-enabled health care technologies refer to a broad range of applications that incorporate AI-based algorithms to support or deliver health care, including clinical decision-support systems, mobile health apps, chatbots, wearable devices, and AI-based diagnostic, screening, and management tools; and (3) focused on research in health care settings. Studies were excluded if they (1) did not use DCE or did not investigate AI-related technologies, (2) were review articles, or (3) were not published in English. This approach ensured the systematic identification of primary research capturing stakeholder preferences toward AI-enabled health care technologies.

### Data Extraction

Two researchers independently extracted detailed data using a standardized data extraction form. The extracted information included study characteristics (publication year, country, sample size, disease area, AI device type, and clinical application), DCE design characteristics (perspective, number of choice sets per block, attributes, and levels, experimental design methods, and econometric analysis methods), as well as the categories of DCE attributes. In addition, study outcome measures were extracted, including attributes identified as the most and least important in the DCE results. Monetary and nonmonetary preference valuation measures reported in the included studies, including willingness to pay (WTP), willingness to save (WTS), and other trade-off estimates, were extracted as originally reported and synthesized descriptively. Any disagreements were resolved through discussion and reexamination of the original articles, and a third reviewer was consulted if necessary.

### Reporting Quality Assessment

Reporting quality was assessed using the DIRECT checklist (Checklist 2) [19], which evaluates 7 reporting domains: purpose and rationale, attributes and levels, experimental design, survey design, sample and data collection, econometric analysis, and reporting of results, comprising a total of 26 items. Each item was graded as “yes” or “no”. Two independent reviewers (XZ and YJ) conducted the assessment, with a third reviewer (SL) resolving any disagreement.

### Synthesis Analysis

A narrative synthesis was conducted to summarize the findings of the included studies. Extracted information was compiled and managed in Microsoft Excel to facilitate comparison across studies. Reported DCE attributes were reviewed and grouped into broader thematic categories to capture the key characteristics. Given the lack of a standardized AI-specific framework, a structured classification system was developed by integrating established health technology assessment (HTA) frameworks, including the HTA Core Model, ISPOR (International Society for Pharmacoeconomics and Outcomes Research) Value Assessment Framework, and NASSS (Non-adoption, Abandonment, Scale-up, Spread, Sustainability) framework. These frameworks were selected due to their widespread use in HTA and shared focus on value-based decision-making and real-world implementation. Attributes were mapped to the integrated framework based on conceptual alignment, and their frequency was calculated according to occurrences in DCE designs. Attributes with potential overlap across domains were independently reevaluated by 2 reviewers based on their conceptual meaning and study context. Discrepancies were resolved through discussion and, if consensus could not be reached, adjudicated by a third reviewer. Attributes that remained conceptually ambiguous were further reviewed through consultation with experts in health preference elicitation, HTA, and AI to ensure consistent and conceptually appropriate domain assignment. Preference patterns were further assessed using relative importance (RI) measures to identify the most and least valued attributes. When RI was not explicitly reported, it was derived from utility coefficients (β) using the utility range method. Specifically, RI was calculated as the range of utility estimates within each attribute (eg, the difference between the highest and lowest β coefficients across attribute levels) divided by the sum of all attribute ranges within the same study. For continuous attributes, RI was calculated by multiplying the regression coefficient by the observed range of attribute levels [20]. Given differences in attribute definitions, coding schemes, and model specifications across studies, RI was used to describe within-study preference structures rather than to enable direct comparison of magnitude across studies.

## Results

### Study Characteristic

**Figure 1.**
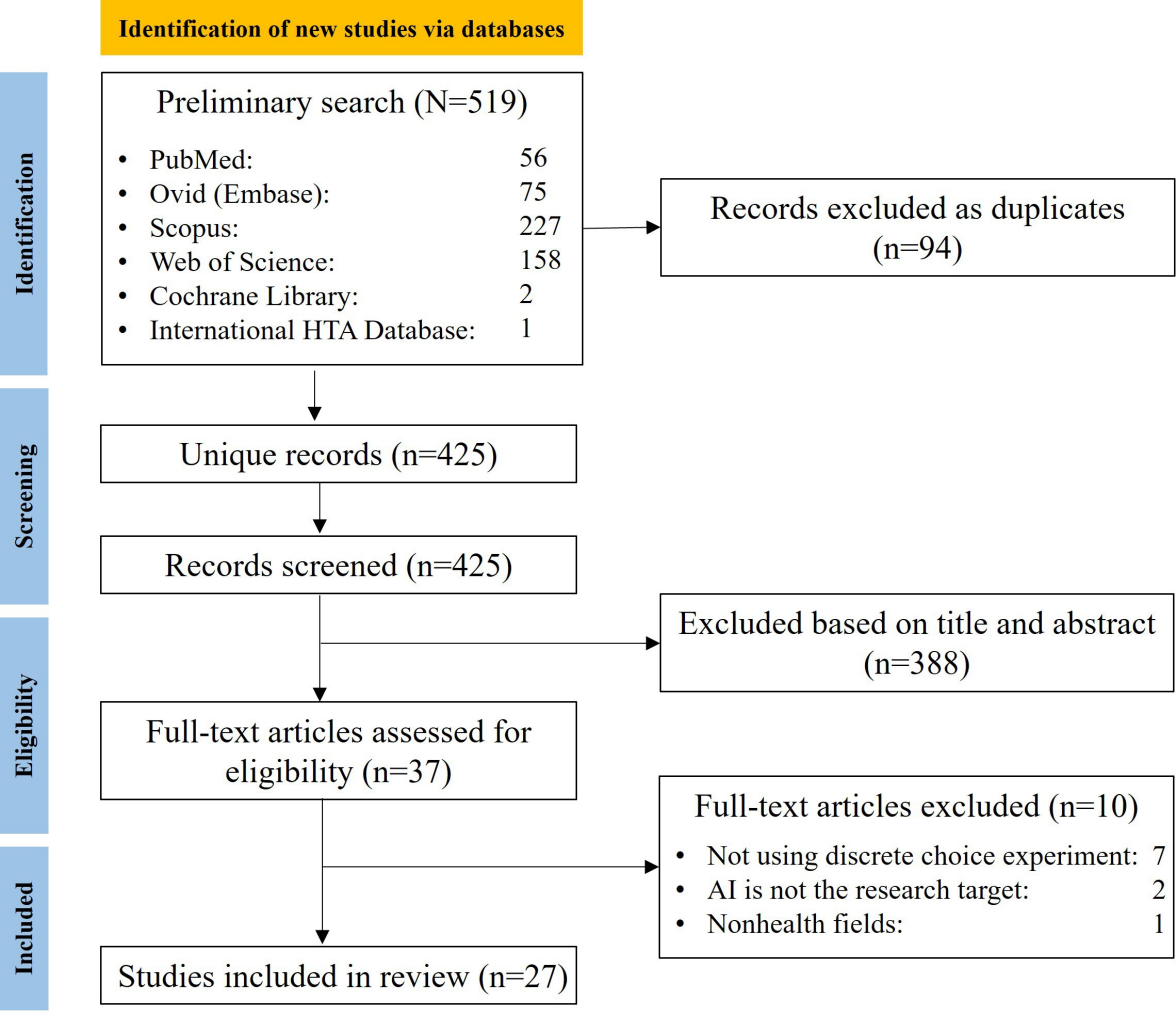
Study selection PRISMA (Preferred Reporting Items for Systematic Reviews and Meta-Analyses) flow diagram. HTA: Health Technology Assessment.

A total of 425 studies were identified. After removing duplicates and screening titles and abstracts, 37 full-text articles were assessed. Of these, 10 were excluded (7 not using DCEs, 2 not focusing on AI technologies, and 1 not in health care). Ultimately, 27 studies comprising 28 DCEs (1 study included 2 DCEs [21]) were included (Figure 1). For methodological and preference analyses, independent DCEs were treated as separate units (n=28). Application-context analyses were conducted at the level of individual AI applications (n=30 application contexts), as some DCEs assessed more than 1 application setting. Attribute analyses were based on all extracted attribute occurrences across included DCEs (n=163 attributes). The studies were published between 2021 and 2026 and conducted across multiple countries, with China (n=7, 25.93%) and Australia (n=6, 22.22%) contributing the most. Sample sizes ranged from 39 to >2000. Most focused on general health services (n=10, 37.04%) or cancer care (n=7, 25.93%), with screening (n=8, 29.63%) and decision support (n=7, 25.93%) as the most common applications. Other contexts included diagnosis and disease management.

Most studies evaluated nontangible AI systems integrated into clinical workflows (n=15, 55.56%), including tools, systems, and programs, while others involved apps, wearable devices, and chatbots. Preferences were mainly elicited from the public (n=16, 59.26%), followed by health care professionals (n=7, 25.93%) and patients (n=5, 18.52%). One study implemented 2 DCEs involving both the public and professionals [22], while another study conducted a single DCE across 4 scenarios [23]. Across the 28 DCEs, the number of attributes ranged from 3 to 8, with 6 being the most common (n=13, 46.43%; Table 1).

**Table 1.** Study characteristics of the included studies.

Study and year	Sample size, n	Country	Diseases	Devices	Clinical application	Perspective	Attributes	Levels	Choice sets^a^, n	DCE^b^ choice design methods	DCE analysis methods
Bankuoru Egala et al [24], 2024	362	Ghana	General health services	Mobile apps	Decision support	Clinicians	6	2‐3	8	Fractional orthogonal design	Hierarchical Bayes model
Haggenmüller et al [25], 2021	728	Germany	Skin cancer	Mobile apps	Diagnosis	Public	7	3‐6	N/A^c^	N/A	Hierarchical Bayes model
Hendrix et al [26], 2021	91	United States	Breast cancer	AI-based systems	Screening	Primary care providers	6	3	15	D-efficiency design	Mixed logit and latent class model
Hendrix et al [21], 2022	66	United States	Breast cancer	AI-based tool	Diagnosis	Radiologists	5	2‐3	8	D-efficiency design	Mixed logit and latent class model
Hendrix et al [21], 2022	66	United States	Breast cancer	AI-based tool	Screening	Radiologists	4	2-3	8	D-efficiency design	Mixed logit and latent class model
Houwen et al [27], 2022	109	Netherlands	Trauma care	Mobile apps	Decision support	Trauma surgeons	6	2‐4	11	D-efficiency design	Conditional and mixed logit model
Jagemann et al [28], 2024	2108	Germany	Mental health	Mobile apps	Treatment	Public	4	3	20	Balanced overlap method	Hierarchical Bayes and mixed logit model
Jagemann et al [29], 2024	126	Germany	Skin cancer	AI-based systems	Screening	Patients	3	4	12	Conjointly algorithm	NA
Kim et al [30], 2025	500	South Korea	Mental health	Chatbot	Treatment	Public	5	2‐3	6	Orthogonal design	Mixed logit model
Latt et al [31], 2024	415	Melbourne	Sexually transmitted infections	AI-based tool	Diagnosis	Public	6	2‐4	6	D-efficiency design	Mixed logit and latent class model
Lewandowska et al [32], 2025	82	Australia	Radiation oncology	AI-based systems	Treatment	Professionals	5	2‐4	12	D-optimal design	Conditional logit model
Lewandowska et al [33], 2025	533	Australia	Radiation therapy	AI-based systems	Treatment	Public	5	2‐4	16	Bayesian D-efficiency design	Mixed logit and latent class model
Lin et al [22], 2022	39; 318	China	Eye disease	AI-based tool	Screening	Medical staff; Residents	7	3‐6	30; 10	N/A	Conditional logit model
Liu et al [34], 2021	767	China	General health services	AI-based program	Diagnosis	Public	6	2‐6	7	Fractional factorial design	Generalized multinomial logit model, mixed logit, and latent class model
Liu et al [35], 2021	528	China	General health services	AI-based program	Diagnosis	Public	6	2‐6	7	Fractional factorial design	Conditional logit and latent class model
Pearce et al [36], 2025	802	Australia	Breast cancer	AI-based program	Screening	Patients	6	3‐4	10	D-efficiency design	Conditional, mixed logit, and latent class model
Ploug et al [37], 2021	1027	Danish	General health services	AI-based services	N/A	Public	6	3	12	Complete enumeration method	Hierarchical Bayes model
Soe et al [38], 2025	411	Australia	Sexually transmitted infections	Mobile apps	Screening	Public	7	2‐4	6	D-efficiency design	Mixed logit and latent class model
Toh et al [39], 2025	596	Singapore	General health services	AI-based systems	Decision support	Public	6	2‐3	NA	NA	Hierarchical Bayes model
Vo et al [23], 2026	585; 592; 559; 566	Australia	Heart disease; Heart disease; Depression; Depression	Mobile apps	Diagnosis; Management; Diagnosis; Management	Public	5	2‐3	5	D-efficiency	Mixed logit and latent class model
von Wedel et al [40], 2022	114	Germany	General health services	AI-based tool	Decision support	Physicians	5	3	10	D-efficiency design	Conditional logit model
Wang et al [41], 2025	957	China	General health services	Chatbots	Decision support	Public	6	2‐5	7	Fractional factorial design	Conditional logit model
Wang et al [42], 2026	396	China	General health services	NA^c^	NA	Patients	6	2	8	Fractional factorial design	Conditional logit model
Woode et al [43], 2025	2063	Australia	Breast cancer	AI-based program	Screening	Public	7	3	9	Balanced overlap design	Conditional and mixed logit model
Xu et al [44], 2025	203	China	Tuberculosis	AI-based program	Management	Patients	6	2‐3	8	D-efficiency design	Mixed logit model
Zhang et al [45], 2025	340	China	General health services	AI-based services	Decision support	Public	6	2‐5	9	Orthogonal design	Mixed logit model
Zheng et al [46], 2025	300	South Africa	General health services	AI-based tool	Decision support	Public	8	2‐3	10	D-efficiency design	Conditional logit model
Zhu et al [47], 2026	340	United States	Atrial fibrillation	Smartwatch or smartphone app	Screening	Patients	8	2	16	D-efficiency design	Mixed effects logistic regression

a Number of choice sets per block.

b DCE: discrete choice experiment.

c N/A: not available.

### Methodological Characteristics of the DCE Studies

#### Experimental Design Characteristics

Of the 27 included studies (28 DCEs), 24 reported the experimental design methods used, yielding a total of 25 DCEs because 1 study conducted 2 separate DCEs with different attribute sets. Among these, statistically efficient designs were most commonly applied, particularly D-efficient or D-optimal designs (n=13, 52%), which are generally considered superior for improving parameter estimation efficiency. Orthogonal or fractional factorial designs were also frequently used (n=7, 28%); however, these traditional approaches are relatively limited in their ability to accommodate complex model structures. The remaining DCEs (n=5, 20%) adopted alternative design strategies, including balanced overlap methods, conjoint algorithms, complete enumeration designs, and Bayesian D-efficient designs. Compared with conventional approaches, Bayesian D-efficient designs offer greater flexibility by incorporating prior information to further optimize statistical efficiency, whereas complete enumeration and simpler overlap methods may be less efficient for parameter estimation.

#### Econometric Models Used

Among the 27 studies (28 DCEs), 14 DCEs applied a single econometric model to analyze the data. Conditional logit models were the most frequently used approach (n=6, 21.43%), followed by hierarchical Bayes models (n=4, 14.29%) and mixed logit models (n=3, 10.71%). In addition, 9 DCEs used multiple analytical models to examine preference heterogeneity or compare model performance. The most common combinations included mixed logit with latent class models (n=7, 25%) and conditional logit with mixed logit models (n=2, 7.41%). Other combinations included conditional logit with latent class models, hierarchical Bayes with mixed logit models, or more complex specifications including generalized multinomial logit models. Overall, preference heterogeneity was explicitly examined in 20 of 28 DCEs (71.43%), primarily using mixed logit (n=15, 53.57%), latent class (n=10, 35.71%), and hierarchical Bayes models (n=5, 17.86%), either alone or in combination. However, analyses of scale heterogeneity were rarely reported (n=1, 3.57%).

### Preferred Attributes of AI Technologies

A 5-domain classification framework was developed through systematic extraction and inductive synthesis, categorizing attributes into AI technology performance, cost, ethical concerns, usability, and clinical applications. The domains of performance, cost, and ethical concerns broadly align with established HTA frameworks (HTA Core Model and ISPOR), with adaptations to reflect AI-specific features such as the “black-box” nature and algorithmic risks. Usability was informed by the NASSS framework, while clinical applications correspond to the “Health Problem and Context” domain, capturing preferences across application settings. Each domain was defined a priori, and detailed operational definitions and examples of included attributes are provided in Table S2 in Multimedia Appendix 1. To minimize overlap, attributes were assigned to a single domain based on their primary conceptual focus as described in the original study context.

**Figure 2.**
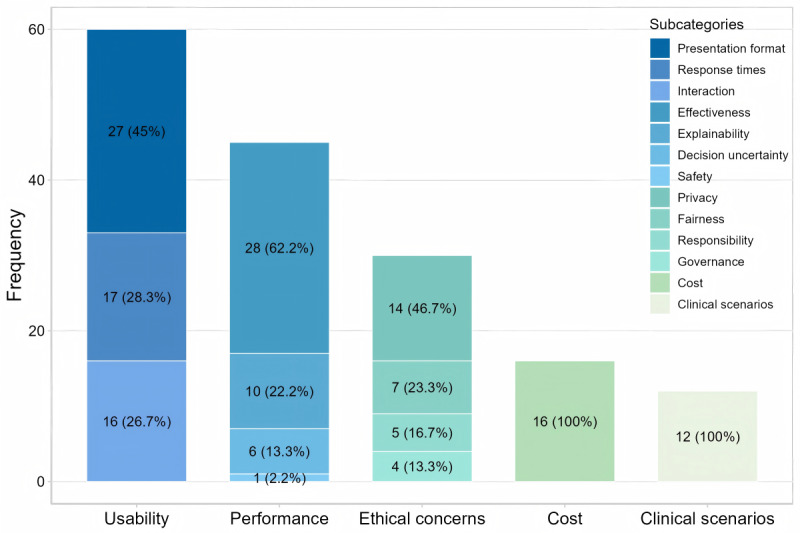
Frequency distribution of discrete choice experiment (DCE) attributes across the included studies by domain and subcategory.

A total of 163 attributes were identified and mapped into 5 attribute domains. Table 2 presents the study-level attributes, including their domain classification and preference rankings (most and least important attributes), whereas Figure 2 summarizes the overall frequency of attribute inclusion across domains and subcategories among all included DCEs. Usability-related attributes were most frequently included (n=60, 36.81%), followed by performance-related attributes (n=45, 27.61%). Within these domains, presentation format (n=27, 45%) and effectiveness (n=28, 62.22%) were the most common subattributes.

Preference outcomes were analyzed at the application context level, with 30 disease-application contexts identified across the included studies. Across all included application contexts, performance attributes were most frequently identified as most important (18/30), particularly effectiveness (17/30), whereas usability attributes were most often rated as least important (14/30), mainly driven by presentation format (9/30). Effectiveness consistently emerged as the most important attribute across stakeholder groups. Clinicians additionally emphasized interaction and response time, while patients and the public showed stronger preferences for cost, with the latter also valuing responsibility. Presentation format was the least important attribute across all groups.

When further stratified by clinical application context, among the 30 disease-application combinations, 1 study (Ploug et al [37]) did not report clinical application and was excluded from this analysis, resulting in 29 contexts. These were categorized into screening (n=8, 27.59%), diagnosis (n=7, 24.14%), decision support (n=7, 24.14%), treatment (n=4, 13.79%), and management (n=3, 10.34%). Performance-related attributes remained dominant in diagnostic (6/7), screening (4/8), treatment (2/4), and management settings (2/3), while decision support systems showed greater variability in attribute prioritization, and usability-related attributes (4/7) were most frequently identified as the most important domain. Usability attributes were consistently ranked as least important in diagnostic, screening (4/8), decision support (3/7), and treatment (3/4) settings, while ethical concerns (2/3) were ranked as least important in management-related contexts.

**Table 2.** Study-level mapping of extracted attributes across framework-based domains and subcategories.

Study	Ethical concerns				Performance				Usability			Cost	Clinical applications	Most preferred attribute	Least preferred attribute
	Privacy	Fairness	Responsibility	Governance	Effectiveness	Safety	Explainability	Decision uncertainty	Interaction	Response times	Presentation format				
Bankuoru Egala et al [24]	1	0	0	0	2	0	0	1	1	1	0	0	0	Interaction	Effectiveness
Haggenmüller et al [25]	3	0	0	0	1	0	1	0	0	1	0	0	1	Effectiveness	Privacy
Hendrix et al [26]	0	1	0	0	2	0	1	1	1	0	0	0	0	Effectiveness	Decision uncertainty
Hendrix et al^a^ [21]	0 0	0 0	0 0	0 0	2 1	0 0	1 0	1 0	0 0	2 2	0 0	0 0	0 0	Effectiveness Effectiveness	Presentation format Presentation format
Houwen et al [27]	0	0	0	1	0	0	0	1	0	0	3	1	0	Presentation Format	Decision uncertainty
Jagemann et al [28]	0	0	0	0	0	0	0	0	1	1	0	1	1	Interaction	Response times
Jagemann et al [29]	0	0	0	0	0	0	0	0	1	1	0	1	0	Interaction	Response times
Kim et al [30]	0	0	0	0	0	0	0	0	1	0	2	1	1	Cost	Presentation format
Latt et al [31]	1	0	0	0	1	0	0	0	0	1	2	1	0	Cost	Presentation format
Lewandowska et al [32]	0	0	0	0	1	0	1	0	1	1	1	0	0	Effectiveness	Presentation format
Lewandowska et al [33]	1	0	0	1	1	0	0	0	0	1	0	1	0	Effectiveness	Privacy
Lin et al [22]	0	0	0	0	2	0	0	0	1	1	1	1	1	Interaction	Presentation format
Liu et al [34]	0	0	0	0	1	0	0	0	1	2	1	1	0	Effectiveness	Response times
Liu et al [35]	0	0	0	0	1	0	0	0	1	2	1	1	0	Effectiveness	Response times
Pearce et al [36]	1	1	1	0	1	0	0	0	1	1	0	0	0	Effectiveness	Privacy
Ploug et al [37]	0	1	1	0	1	0	1	0	0	0	0	0	2	Responsibility	Severity
Soe et al [38]	0	0	0	0	1	0	0	0	1	1	3	1	0	Cost	Presentation format
Toh et al [39]	0	1	1	0	1	0	1	0	0	0	0	0	2	Responsibility	Clinical applications
Vo et al^b^ [23]	1	0	0	0	1	0	1	0	1	0	0	1	0	Effectiveness (all 4 scenarios)	Privacy (all 4 scenarios)
von Wedel et al [40]	0	0	0	0	1	0	0	0	0	1	1	1	1	Response times	Interaction
Wang et al [41]	1	0	0	0	1	0	1	0	0	0	1	1	1	Effectiveness	Privacy
Wang et al [42]	1	1	1	0	1	0	1	1	0	0	0	0	0	Effectiveness	Explainability
Woode et al [43]	0	1	1	0	2	0	0	1	1	1	0	0	0	Effectiveness	Decision uncertainty
Xu et al [44]	1	0	0	0	0	0	0	0	1	1	1	1	1	Cost	Clinical applications
Zhang et al [45]	0	0	0	0	1	0	1	0	0	0	3	1	0	Effectiveness	Presentation format
Zheng et al [46]	2	0	0	0	0	0	1	0	0	0	3	1	1	Explainability	Presentation format
Zhu et al [47]	1	1	0	2	2	1	0	0	1	0	0	0	0	Governance	Safety

a The study implemented 2 discrete choice experiments.

b The study conducted 1 discrete choice experiment across 4 scenarios, yielding 4 observations each for the most preferred and least preferred attributes.

### Willingness to Pay and Trade-Off Estimates

Ten (37.04%) studies quantified the economic value of DCE attributes through monetary WTP (n=8) or WTS (n=2), with time as the trade-off metric. The highest and lowest marginal WTP within each study are extracted in Table 3. Among the 7 studies using monthly or per-visit cost metrics, the highest WTP was associated with performance and usability attributes. Participants were willing to pay an additional US $0.08 to US $0.26 for every 1% increase in diagnostic accuracy, US $2.24-US $3.25 for AI interactions, and up to US $79 for reduced decision uncertainty. Conversely, the lowest WTP was associated with usability (presentation format) and ethical concerns, ranging from US $0.02 to US $2. Reflecting an annual cost scale, one study reported higher baseline values, peaking at US $29,244.8 for outcome integration (performance) and dropping to US $4162.6 for local information (ethical concerns). Two studies reported trade-offs using WTS, and the highest WTS was consistently associated with performance, for 0.7 minutes per 1% increase in accuracy and 111 minutes for maintaining current diagnostic accuracy. In contrast, the lowest WTS was observed for decision uncertainty and ethical concerns, at 32 and 0 minutes, respectively.

**Table 3.** The highest and lowest willingness-to-pay (WTP) or willingness to save or wait (WTS) estimates across the included studies.

Study	Country	Attributes (max)	WTP (max)	Attributes (least)	WTP (least)
Monetary willingness to pay					
Houwen et al [27]	Netherlands	Patient-reported outcome measures	€22,496 (US $29,244.8)	Local information adjustment (reference: own hospital logo)	€3202 (US $4162.6)
Kim et al [30]	South Korea	Emotion (facial expression and empathetic response; reference: no expression)	US $2.31 (per month)	Image (the chatbot appears similar to a human; reference: the chatbot appears similar to a robot)	US $0.625 (per month)
Lewandowska et al [33]	Australia	Diagnosis and treatment oversight assistive (base=autonomous)	US $79	AI-driven use of your data (base=no consent required)	US $2
Liu et al [35]	China	2017: AI and clinician diagnosis method (reference: clinician) 2020: diagnosis accuracy	2017: US $2.24 (per 1% increase) 2020: US $0.26 (per 1% increase)	2017: diagnosis time (minutes) 2020: diagnosis time (minutes)	2017: US $0.09 (decrease time for per minute) 2020: US $0.01 (decrease time for per minute)
von Wedel et al [40]	Germany	High time savings (reference: low time savings)	€5.16 (US $5.43)	Provider modality manufacturer (reference: RIS/PACS software provider)	€0.02 (US $0.02)
Xu et al [44]	China	Service provider: AI+physician (reference: AI)	￥23.01 (US $3.25)	Service frequency monthly (reference: weekly)	￥2.01 (US $0.28)
Wang et al [41]	China	Information utility very practical (reference: very impractical)	￥11.451 (US $1.62)	Not to allow the collection of background information (reference: allow)	￥0.839 (US $0.12)
Zhang et al [45]	China	100% symptom-specific results (reference: 60%)	￥24.01 (US $3.39)	General linguistic comprehensibility (reference: difficult)	￥0.83 (US $0.85)
Willingness to save or wait time					
Lewandowska et al [32]	Australia	Accuracy same as current (reference: reduce)	111 min	Assistive (base=autonomous)	32 min
Pearce et al [36]	Australia	Accuracy	0.7 min (per 1% increase in accuracy)	Privacy (improve breast screen or research) (base=direct medical care only)	0 min

### Reporting Quality Assessment

**Figure 3.**
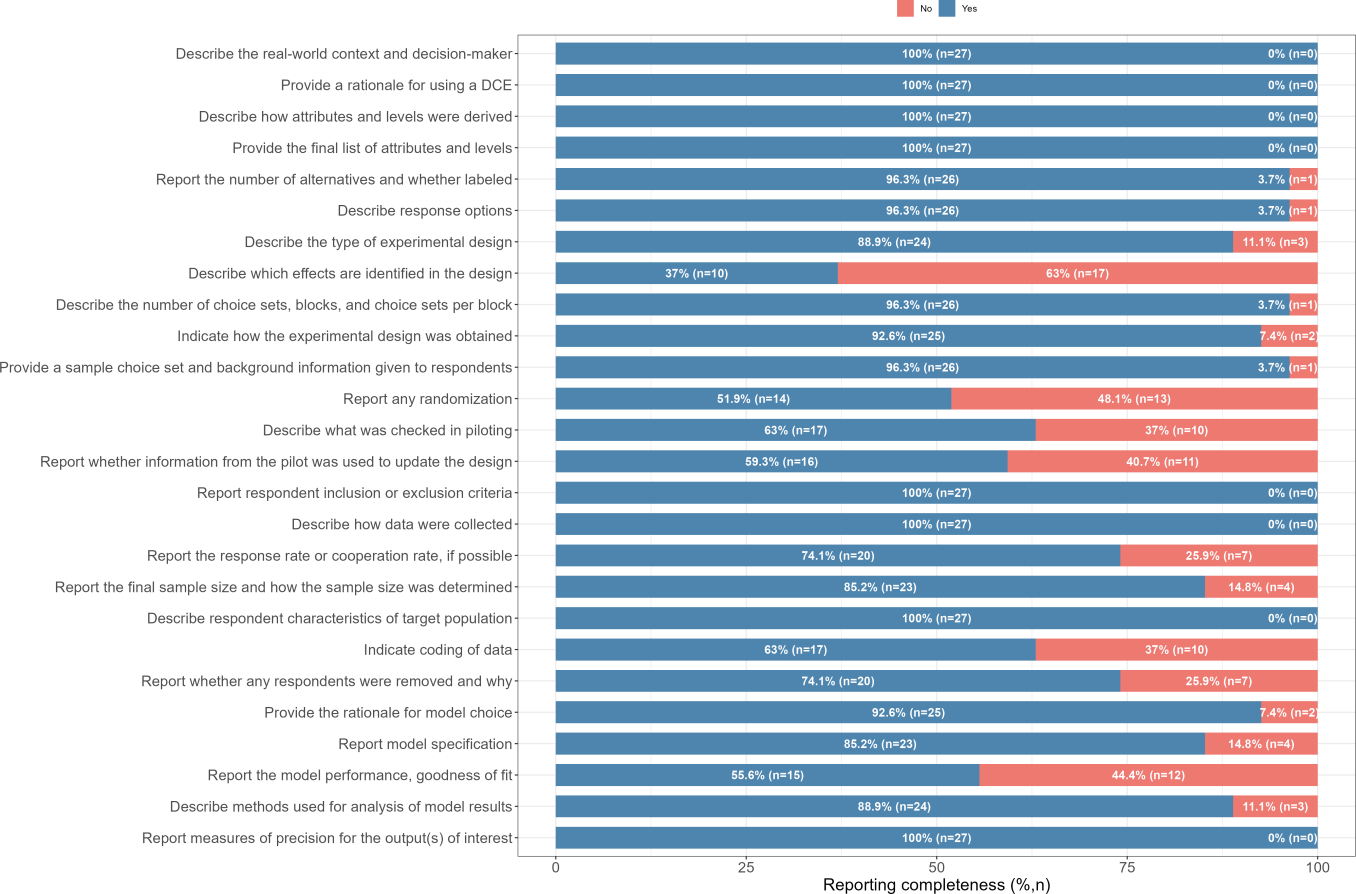
Proportion of studies reporting each item in the DIRECT (DIscrete choice experiment REporting ChecklisT). DCE: discrete choice experiment.

Reporting quality was assessed using the DIRECT checklist (Figure 3). Across the 27 studies, the mean completion rate for the 26 items was 84.47% (SD 11.49%), ranging from 50% to 100%. Four studies achieved complete reporting (100%), 2 of which explicitly referenced the DIRECT checklist.

Among the 7 reporting domains, “Purpose and rationale” and “Attributes and levels” were fully reported in all studies (n=27, 100%), while the “Sample and data collection” reporting domain also showed high completeness (n= 25, 92.59%). In contrast, several items were less consistently reported. The lowest reporting rate was observed for item 8 (identification of design effects) in the experimental design domain (n=10, 37.04%). The reporting of randomization in the survey design domain was also limited (n=14, 51.85%). Other less frequently reported items included model performance (n=15, 55.56%) and the use of pilot study information (n=17, 62.96%). Overall, although reporting quality was generally high, items related to experimental design and survey procedures were less consistently reported. Detailed results are provided in Checklist 2.

## Discussion

### Principal Findings

This review provides the first comprehensive synthesis of DCEs on stakeholder preferences for AI-enabled health care technologies, with a marked increase in studies since 2021. Across different stakeholders, effectiveness consistently emerged as the primary driver of preferences, underscoring the importance of performance in the acceptance of AI in health care. While reporting completeness was generally high, the findings suggest that further improvements are needed to better align study designs with stakeholder priorities.

### Stakeholder Preferences and Attribute Priorities

The consistent prioritization of effectiveness across stakeholder groups suggests that clear clinical benefit is essential for the acceptance of AI-enabled health care technologies [30,34,35]. This may be due to ongoing uncertainty about their real-world performance and reliability. Across the included DCE studies, differences in the most important attributes appear to reflect role-specific concerns. Clinicians tend to value interaction and response time, likely because AI systems need to fit into clinical workflows without increasing the time burden [48]. In contrast, patients and the public place more emphasis on cost, reflecting concerns about financial burden and value [49]. Although effectiveness was most frequently identified as the dominant attribute, this finding should not be interpreted as indicating a universal preference across AI-enabled health care technologies. Our review suggests that preference structures vary according to application context. Performance-related attributes were consistently prioritized in diagnosis, treatment, and disease management, whereas usability assumed greater importance in decision-support applications. These findings indicate that the relative importance of AI attributes is shaped by the intended use of the technology rather than stakeholder type alone. Other contextual factors, including AI functionality, target population, and health care setting, are also likely to influence preferences, although current evidence remains insufficient to evaluate their effects systematically. Future DCEs should examine these sources of contextual heterogeneity to improve the generalizability and applicability of preference evidence.

This study suggests that attributes commonly included in AI-related DCE designs may not fully align with those most strongly valued by stakeholders. Usability-related attributes are frequently incorporated but are often of limited importance, whereas effectiveness consistently emerges as the primary driver. This pattern is consistent with prior AI-related DCE evidence, such as studies in screening contexts where usability-related attributes, such as interaction, are commonly included [18]. However, compared with traditional health care DCEs, where attribute selection and preference evaluation have generally focused more consistently on performance-related dimensions such as clinical outcomes, AI-related DCEs appear to place greater emphasis on usability and process-oriented attributes [50-52]. This pattern was also reflected in WTP findings, where higher monetary or time trade-offs were more frequently associated with performance-related improvements, particularly diagnostic accuracy and reduced uncertainty, whereas presentation-related attributes generally attracted lower values. AI-related DCEs appear to place greater emphasis on usability and process-oriented features at the design stage. This suggests a limitation in current DCE practice in AI-enabled health care, where attribute selection may not adequately reflect stakeholder priorities [53].

Additionally, the lower importance of usability attributes may partly reflect design-related effects in some studies. In DCEs, attribute importance depends on both attribute selection and the utility variation implied by attribute levels. For example, in a study by Liu et al [34], usability attributes, such as response time, were defined using relatively narrow level ranges (eg, diagnosis time categories of 0, 15, and 30 minutes), compared with wider ranges for performance attributes (eg, diagnostic accuracy from 60%-100%), which may reduce observed utility differences and attenuate their estimated importance. These design factors should be considered when interpreting DCE-based preference evidence. Importantly, observed differences across stakeholder groups may also reflect variation in study context, attribute selection, AI application type, and sample composition, rather than solely true stakeholder-level preference heterogeneity.

### Reporting Quality and Methodological Implications

Mean reporting completeness was relatively high (84.47%, SD 11.49%), consistent with prior AI-related health economic reviews reporting rates of 66% to 77.4% [54-56]. However, key methodological elements, particularly those related to experimental design, were insufficiently reported. For example, the identification of design effects (n=10, 37.04%) and the reporting of randomization procedures (n=14, 51.85%) were limited, aligning with previous findings of substantial gaps in analytical planning, with reporting rates as low as 0% and 10.52% [54,55]. Similar reporting deficiencies have also been identified in non–AI-related DCE reviews, where the ISPOR checklist was applied to evaluate reporting quality and revealed insufficient reporting of key experimental design elements [57,58]. It is important to emphasize that the DIRECT checklist assesses reporting completeness rather than methodological quality. Accordingly, missing reporting does not imply that key design features such as randomization, pilot testing, design-effect assessment, or model diagnostics were not undertaken in the original studies. Rather, incomplete reporting limits the ability to fully appraise the link between study design and estimated preferences, which may reduce confidence in the interpretation of trade-offs and WTP estimates [59]. This issue may be further exacerbated in AI-related DCEs, where increasing model complexity may outpace reporting transparency, thereby constraining the evaluation of methodological justification [60,61].

Model performance was reported in only 55.56% of the studies, restricting the evaluation of model fit and comparison across specifications. However, this finding reflects limitations in reporting rather than direct evidence of inadequate model performance or inappropriate model selection. Most studies relied on conditional and mixed logit models, with limited use of multiple models to assess robustness or explore heterogeneity. While some studies accounted for preference heterogeneity, scale heterogeneity was rarely addressed [34,59,62]. This is particularly relevant in AI contexts, where system-embedded and less observable technologies may increase the difficulty of attribute interpretation, leading to greater response variability. As a result, observed variation may partly reflect response noise rather than true preference heterogeneity, potentially biasing results [60,61,63]. The use of more flexible and complementary modeling approaches may improve robustness [64-66].

### Implications of the Attribute Classification Framework

In the absence of an AI-specific evaluation framework, we integrated the HTA Core Model, ISPOR Value Assessment Framework, and NASSS framework to categorize attributes. HTA and ISPOR capture clinical, economic, and value dimensions, while NASSS addresses the sociotechnical complexities of technology adoption [67-69]. This integrated approach is well suited to AI, as it incorporates features such as limited explainability, decision uncertainty, and algorithmic fairness that extend beyond traditional domains. Although AI-related DCEs share similarities with conventional health care studies, they additionally reflect interaction dynamics and “black-box” concerns. By combining value assessment with implementation complexity, this framework provides a structured and relevant basis for synthesizing DCE evidence and informing the design of AI-enabled health care services.

These findings also have implications for health care decision-making. For developers and health care providers, understanding which AI attributes are most valued may support user-centered technology design and implementation strategies. For regulators, payers, and HTA agencies, preference evidence may help identify technology characteristics that are most relevant to stakeholder acceptance and real-world uptake. Incorporating stakeholder preferences into the evaluation of AI-enabled health care technologies may therefore facilitate more patient-centered and context-sensitive decision-making.

### Limitations

Several limitations should also be noted. First, the proposed attribute classification framework, although informed by the HTA Core Model, ISPOR, and NASSS frameworks, may not fully capture all relevant dimensions of AI-enabled health care. In addition, as the framework is derived from attributes reported in existing DCE studies, it reflects what has been operationalized in preference research rather than the full spectrum of characteristics of AI-based health care services. Second, reporting quality was assessed using the DIRECT checklist, which focuses on transparency rather than methodological quality. As such, while reporting gaps can be identified, this approach may not fully capture all sources of bias in study design and analysis. Third, only English-language publications were included, which may have resulted in the omission of relevant studies published in other languages. Finally, this review synthesizes stakeholder preferences based on DCE evidence. Although DCEs are well suited for eliciting trade-offs, they represent only 1 preference elicitation approach. Moreover, all included studies elicited stated preferences using hypothetical choice scenarios, and none examined the relationship between stated preferences and may not fully reflect real-world decision-making, particularly for complex and less observable AI technologies. Nevertheless, this review provides a structured synthesis of stakeholder preferences and reporting practices in AI-related DCEs. Future research should expand attribute identification beyond existing DCE designs and adopt complementary methods to better capture the complexity and real-world characteristics of AI-enabled health technologies.

### Conclusions

This review identifies a mismatch between DCE attribute design and stakeholder preferences in AI-enabled health care. Effectiveness generally emerges as a key determinant of preferences, particularly in clinically oriented settings, while usability and performance attributes vary in importance across AI applications. Preference structures are context-dependent rather than uniform across technologies. Although overall reporting completeness was high, key methodological elements such as design effects and randomization were inconsistently reported, indicating room for improvement in study design and reporting transparency. Strengthening methodological rigor and aligning attribute selection with decision contexts may enhance the interpretability of DCE evidence and support the development of AI technologies that better reflect real-world health care decision needs.

## Supplementary material

10.2196/103197 Multimedia Appendix 1 Search strategies for different databases and domain definitions and attribute mapping rules.

10.2196/103197 Checklist 1 PRISMA 2020 checklist.

10.2196/103197 Checklist 2 DIRECT checklist.

## Abbreviations

DCE: discrete choice experiment
DIRECT: DIscrete choice experiments REporting ChecklisT
HTA: Health Technology Assessment
ISPOR: International Society for Pharmacoeconomics and Outcomes Research
NASSS: Non-adoption, Abandonment, Scale-up, Spread, Sustainability
PRISMA: Preferred Reporting Items for Systematic Reviews and Meta-Analyses
PROSPERO: International Prospective Register of Systematic Reviews
RI: relative importance
WTP: willingness to pay
WTS: willingness to save or wait
