# Supplementary material for: Preferences for AI-Enabled Health Care Technologies: Systematic Review of Discrete Choice Experiments and Reporting Quality Assessment Using the DIRECT Checklist
Source: J Med Internet Res. 2026 Aug 10;28:e103197. doi: 10.2196/103197 (PMC13455583; doi:10.2196/103197)
Supplement: Supplementary file 1 [file jmir-v28-e103197-s001.docx]

Table S1. Search strategy from different database. (2026.03.19)

**Scopus**

| Search | Query | Items |
| --- | --- | --- |
| #1 | TITLE-ABS-KEY ("artificial intelligence" OR AI OR "machine intelligence" OR "computational intelligence" OR "computer reasoning" OR "knowledge acquisition" OR "knowledge representation" OR "computer vision system*" OR "computer vision") | 1,373,063 |
| #2 | TITLE-ABS-KEY ("discrete choice experiment*" OR "discrete choice model*" OR "choice-based conjoint" OR "conjoint analysis" OR "stated preference*") | 23,045 |
| #3 | #1 AND #2 | 227 |

**Pubmed**

| Search | Query | Items |
| --- | --- | --- |
| #1 | (((((((((((((((((((Artificial Intelligence[MeSH Terms]) OR (Intelligence, Artificial[Title/Abstract])) OR (Computer Reasoning[Title/Abstract])) OR (Reasoning, Computer[Title/Abstract])) OR (AI (Artificial Intelligence[Title/Abstract]))) OR (Machine Intelligence[Title/Abstract])) OR (Intelligence, Machine[Title/Abstract])) OR (Computational Intelligence[Title/Abstract])) OR (Intelligence, Computational[Title/Abstract])) OR (Computer Vision Systems[Title/Abstract])) OR (Computer Vision System[Title/Abstract])) OR (System, Computer Vision[Title/Abstract])) OR (Systems, Computer Vision[Title/Abstract])) OR (Vision System, Computer[Title/Abstract])) OR (Vision Systems, Computer[Title/Abstract])) OR (Knowledge Acquisition (Computer[Title/Abstract]))) OR (Acquisition, Knowledge (Computer[Title/Abstract]))) OR (Knowledge Representation (Computer[Title/Abstract]))) OR (Knowledge Representations (Computer[Title/Abstract]))) OR (Representation, Knowledge (Computer[Title/Abstract])) | 327096 |
| #2 | ((((discrete choice experiment[Title/Abstract]) OR (discrete choice model[Title/Abstract])) OR (choice-based conjoint[Title/Abstract])) OR (conjoint analysis[Title/Abstract])) OR (stated preference[Title/Abstract]) | 5,648 |
| #3 | #1 AND #2 | 56 |

**Web of Science**

| Search | Query | Items |
| --- | --- | --- |
| #1 | TS= ("artificial intelligence" OR AI OR "machine intelligence" OR "computational intelligence" OR "computer reasoning" OR "knowledge acquisition" OR "knowledge representation" OR "computer vision system*" OR "computer vision") | 628,109 |
| #2 | TS= ("discrete choice experiment*" OR "discrete choice model*" OR "choice-based conjoint" OR "conjoint analysis" OR "stated preference*") | 21,791 |
| #6 | #2 AND #1 | 158 |

**Ovid**

| Search | Query | Items |
| --- | --- | --- |
| #1 | ("artificial intelligence" or AI or "machine intelligence" or "computational intelligence" or "computer reasoning" or "knowledge acquisition" or "knowledge representation" or "computer vision system*" or "computer vision").mp. [mp=title, abstract, heading word, drug trade name, original title, device manufacturer, drug manufacturer, device trade name, keyword heading word, floating subheading word, candidate term word] | 260,881 |
| #2 | ("discrete choice experiment*" OR "discrete choice model*" OR "choice-based conjoint" OR "conjoint analysis" OR "stated preference*)"[mp=title, abstract, heading word, drug trade name, original title, device manufacturer, drug manufacturer, device trade name, keyword heading word, floating subheading word, candidate term word] | 8,709 |
| #3 | #1 AND #2 | 75 |

**Cochrane Library**

| Search | Query | Items |
| --- | --- | --- |
| #1 | MeSH descriptor: [Artificial Intelligence] explode all trees | 3,997 |
| #2 | ("discrete choice experiment" OR "discrete choice model" OR "choice-based conjoint" OR "conjoint analysis" OR "stated preference"):ti,ab,kw | 534 |
| #3 | #1 AND #2 | 2 |

**International Health technology assessment (HTA) database**

| Search | Query | Items |
| --- | --- | --- |
| #1 | "Artificial Intelligence"[mh] | 52 |
| #2 | All ("discrete choice experiment" OR "discrete choice model" OR "choice-based conjoint" OR "conjoint analysis" OR "stated preference") | 17 |
| #3 | #1 AND #2 | 1 |

Table S2. Domain definitions and attribute mapping rules

| Domain | Subdomain | Operational definition | Representative attributes | Classification evidence |
| --- | --- | --- | --- | --- |
| Performance | Effectiveness | The extent to which AI achieves intended clinical or operational outcomes | Diagnostic accuracy, prediction accuracy, treatment effectiveness, symptom-specific results | [1,2,3] |
|  | Safety | Potential risks or adverse consequences associated with AI use | Misdiagnosis risk, false-positive rate, false-negative rate | [1,2,4] |
|  | Explainability | Ability of users to understand how AI generates recommendations or outputs | Information utility, linguistic comprehensibility, interpretability of results | [1,2,5] |
|  | Decision uncertainty | Degree of confidence and reliability in AI-supported decisions | Probability of error, uncertainty in recommendations, level of clinician oversight | [1,2,6] |
| Usability | Interaction | Features influencing user-AI communication and workflow integration | Human-AI collaboration, service provider, clinician involvement, chatbot empathy | [7,8] |
|  | Response time | Timeliness and efficiency of AI-generated outputs | Waiting time, diagnosis time, consultation time saved | [7,8] |
|  | Presentation format | How AI information or recommendations are displayed to users | Interface design, visual format, chatbot appearance, provider modality | [7,8] |
| Ethical concerns | Privacy | Protection and use of personal data | Data collection, data sharing, consent requirements | [1,2,9] |
|  | Fairness | Equity and absence of discriminatory outcomes | Algorithmic bias, equal treatment across groups | [1,2,9,10] |
|  | Responsibility | Accountability for AI-generated decisions and outcomes | Liability attribution, human oversight responsibilities | [1,2,11] |
|  | Governance | Regulatory and institutional mechanisms overseeing AI use | Certification, regulation, governance framework | [1,2,12] |
| Cost | — | Financial burden associated with AI adoption or use | Out-of-pocket payment, subscription fee, service cost | [1,2,13] |
| Clinical applications | — | Context in which AI is applied, including disease area, care setting, or intended use | Screening, diagnosis, treatment planning, hospital setting | [7] |

**Reference**

1.	Kristensen FB, Lampe K, Wild C, Cerbo M, Goettsch W, Becla L. The HTA Core Model(®)-10 Years of Developing an International Framework to Share Multidimensional Value Assessment. Value Health. 2017 Feb;20(2):244-50. PMID: 28237203. doi: 10.1016/j.jval.2016.12.010.

2.	Lakdawalla DN, Doshi JA, Garrison LP, Jr., Phelps CE, Basu A, Danzon PM. Defining Elements of Value in Health Care-A Health Economics Approach: An ISPOR Special Task Force Report [3]. Value Health. 2018 Feb;21(2):131-9. PMID: 29477390. doi: 10.1016/j.jval.2017.12.007.

3. Ouanes K, Farhah N. Effectiveness of Artificial Intelligence (AI) in Clinical Decision Support Systems and Care Delivery. J Med Syst. 2024 Aug 12;48(1):74. PMID: 39133332. doi: 10.1007/s10916-024-02098-4.

4.	Niemiec E. Will the EU Medical Device Regulation help to improve the safety and performance of medical AI devices? Digit Health. 2022 Jan-Dec;8:20552076221089079. PMID: 35386955. doi: 10.1177/20552076221089079.

5. Shin D. The effects of explainability and causability on perception, trust, and acceptance: Implications for explainable AI. International Journal of Human-Computer Studies. 2021 2021/02/01/;146:102551. doi: https://doi.org/10.1016/j.ijhcs.2020.102551.

6.	Harish V, Morgado F, Stern AD, Das S. Artificial Intelligence and Clinical Decision Making: The New Nature of Medical Uncertainty. Acad Med. 2021 Jan 1;96(1):31-6. PMID: 32852320. doi: 10.1097/acm.0000000000003707.

7..	Greenhalgh T, Wherton J, Papoutsi C, Lynch J, Hughes G, A'Court C, et al. Beyond Adoption: A New Framework for Theorizing and Evaluating Nonadoption, Abandonment, and Challenges to the Scale-Up, Spread, and Sustainability of Health and Care Technologies. J Med Internet Res. 2017 Nov 1;19(11):e367. PMID: 29092808. doi: 10.2196/jmir.8775

8. Biswas, P. (2026). Intelligent User Interface: Usable Artificial Intelligence and Artificial Intelligence for Usability (1st ed.). CRC Press. <https://doi.org/10.1201/9781003646716>

9. Fairness and Privacy: Sorathiya A, Ginde G. Exploring ethical concerns of mobile applications from App Reviews: A literature survey. Journal of Systems and Software. 2026:112900.

10. Culyer AJ, Bombard Y. An equity framework for health technology assessments. Med Decis Making. 2012 May-Jun;32(3):428-41. PMID: 22065143. doi: 10.1177/0272989x11426484.

11. Huang C, Zhang Z, Mao B, Yao X. An overview of artificial intelligence ethics. IEEE transactions on artificial intelligence. 2022;4(4):799-819.

12. Goverance: Xue L, Pang Z. Ethical governance of artificial intelligence: An integrated analytical framework. Journal of Digital Economy. 2022 2022/06/01/;1(1):44-52. doi: 10.1016/j.jdec.2022.08.003.

13. Cost: Neumann PJ, Sanders GD, Russell LB, Siegel JE, Ganiats TG. Cost-effectiveness in health and medicine: Oxford University Press; 2016. ISBN: 0190492945.
